# Supplementary material for: Histological Correlation between Tonsillar and Glomerular Lesions in Patients with IgA Nephropathy Justifying Tonsillectomy: A Retrospective Cohort Study
Source: Int J Mol Sci. 2024 May 13;25(10):5298. doi: 10.3390/ijms25105298 (PMC11120689; doi:10.3390/ijms25105298)
Supplement: Supplementary file 1 [file ijms-25-05298-s001.zip › ijms-2965951-supplementary.pdf]

Supplementary Table S1. **S**taining conditions used for palatine tonsil and kidney.

Tissue	Section	Antibody type	Clonality	Animal	Clone	Dilution	Enhancement	Supplier
palatine tonsil	paraffine-embedded	HLA-DP, DQ, DR	monoclonal	mouse	M0775	1,000	none	DAKO
palatine tonsil	paraffine-embedded	CD3	monoclonal	mouse	413241	2	CC1 64min	Nichirei Bioscience
palatine tonsil	paraffine-embedded	CD4	monoclonal	rabbit	790-4423	1	CC1 64min	VENTANA
palatine tonsil	paraffine-embedded	CD8	monoclonal	mouse	M7103	25	CC1 36min	DAKO
palatine tonsil	paraffine-embedded	CD20(L26)	monoclonal	mouse	412441	1	CC1 36min	Nichirei Bioscience
palatine tonsil	paraffine-embedded	CD34	monoclonal	mouse	413111	2	CC1 36min	Nichirei Bioscience
palatine tonsil	paraffine-embedded	CD208	polyclonal	rabbit	10527-RP02	100	CC1 37min	Sino Biological

palatine tonsil	paraffine-embedded	cytokeratin (AE1/AE3)	monoclonal	mouse	412811	1	proteinase 8 min	Nichirei Bioscience
palatine tonsil	paraffine-embedded	D2-40	monoclonal	mouse	413451	1	none	Nichirei Bioscience
palatine tonsil	paraffine-embedded	IgG	monoclonal	mouse	413271	10	proteinase 32 min	Nichirei Bioscience
palatine tonsil	paraffine-embedded	IgA	polyclonal	rabbit	413581		proteinase 12min	Nichirei Bioscience
kidney	frozen	FITC-labeled IgG	monoclonal	mouse	760-2680			VENTANA
kidney	frozen	FITC-labeled IgA	monoclonal	mouse	760-2681			VENTANA
kidney	frozen	FITC-labeled IgM	monoclonal	mouse	760-2682			VENTANA
kidney	frozen	FITC-labeled C3	monoclonal	mouse	760-2686			VENTANA
kidney	frozen	FITC-labeled C1q	monoclonal	mouse	760-2688			VENTANA

HLA, human leukocyte antigen; CD, cluster of differentiation; FITC, fluorescein isothiocyanate; min., minutes, CC1, cell conditioning 1

(**a**) (**b**)

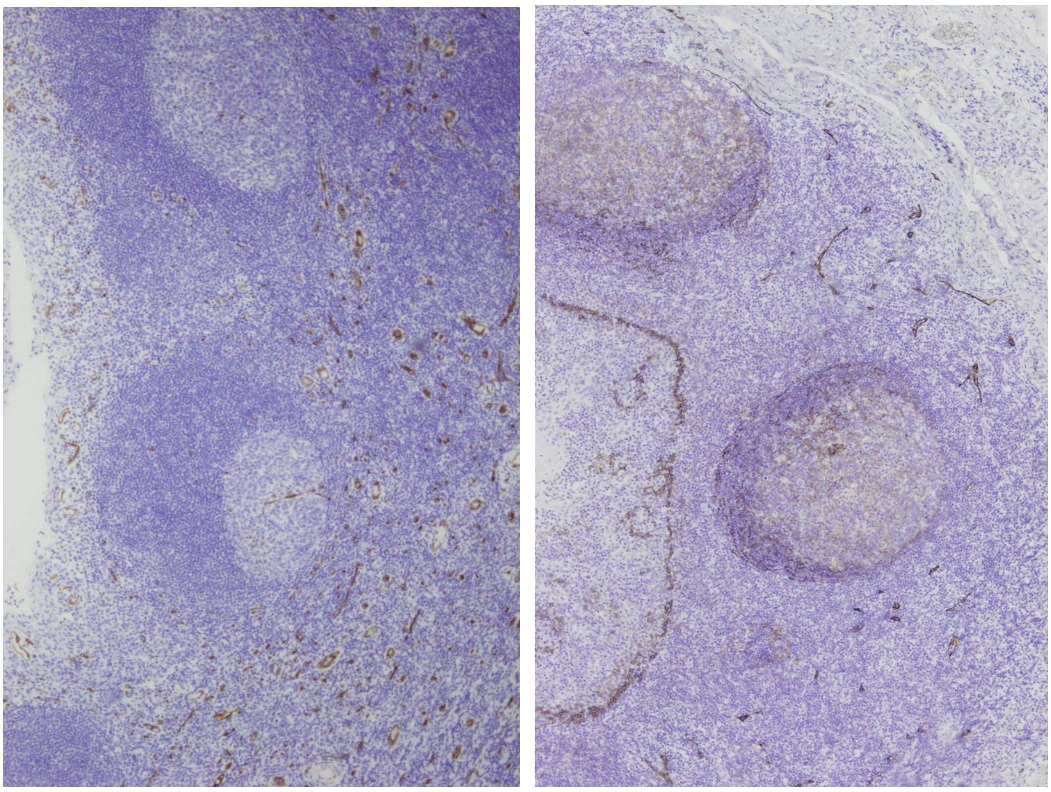

Supplementary Figure S1. Immunostaining conditions for evaluating high epithelial venule (HEV) and lymphatic vessels in palatine tonsils. HEVs were stained with anti-CD 34 antibodies and were distributed in the areas of T-nodules (**a**). Lymphatic vessels were stained with anti-D2-40 antibodies and were concentrated in the same areas of T-nodules (**b**).

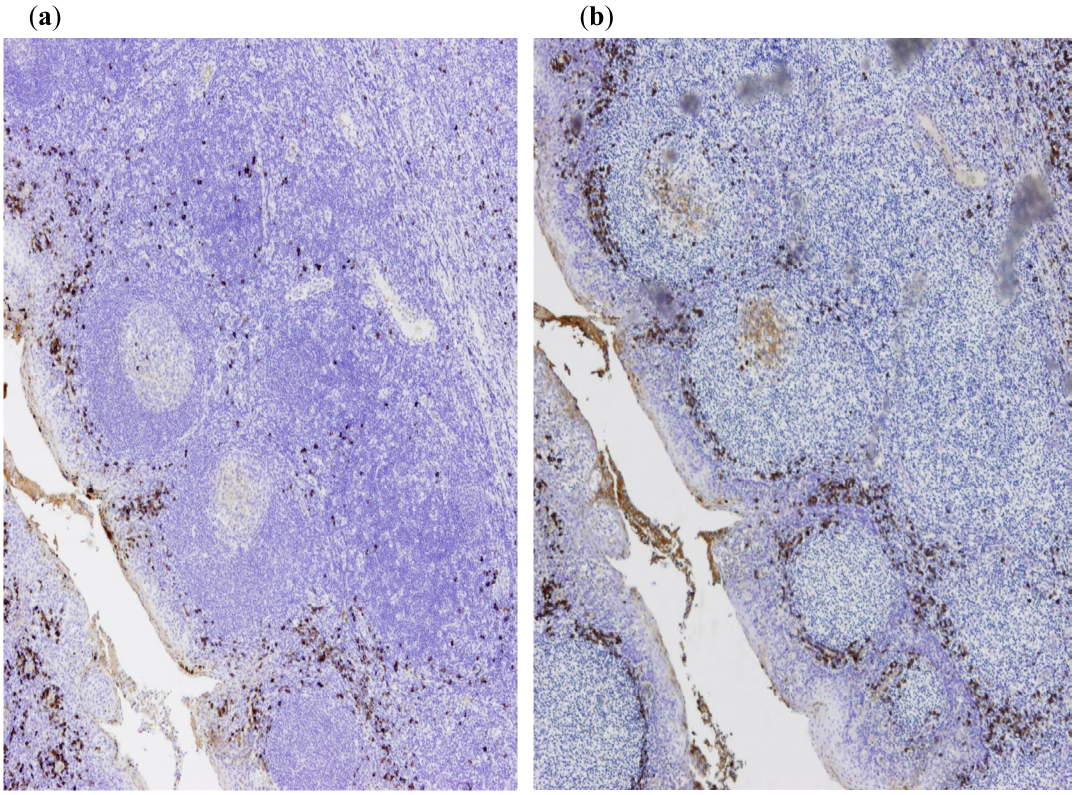

Supplementary Figure S2. Immunostaining of IgA- and IgG-bearing plasma cells in TON-A. IgA- and IgG-bearing plasma cells were distributed around the mantle zone of lymphatic follicles beneath the tonsillar crypt epithelium (Immunostaining of IgA-bearing cells : Figure S2 a, Immunostaining of IgG-bearing cells : Figure S2 b)

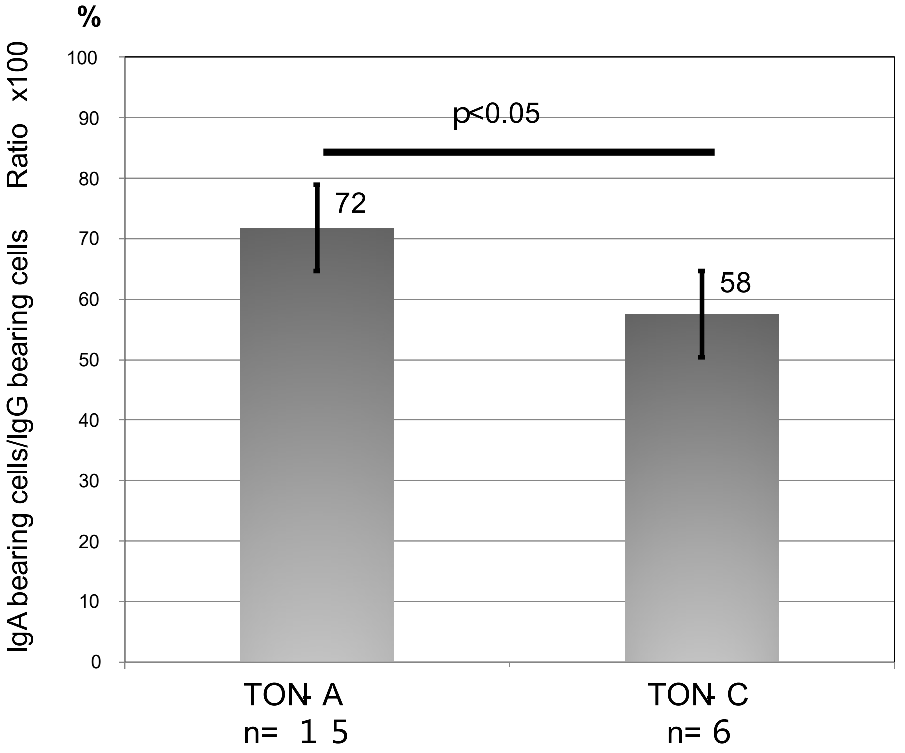

Supplementary Figure S3. Statistical comparison of TON-A and TON-C concerning the ratio of IgA-/IgG-bearing cells around lymphatic follicles. Overall, 15 and 6 cases of TON-A and TON-C were stained with anti-IgG and anti-IgA antibodies, respectively. Five locations (0.8 mm^2 per location) for estimating the frequencies of IgA- and IgG-bearing plasma cells were counted by an image analyzer (NIS Elements D 3.1,) and the ratios of IgA-/IgG-bearing plasma cells of TON-A and TON-C were compared. In TON-A, the numbers of IgA- and IgG-bearing plasma cells composed 121 + −54/0.8 and 182 + − 74/08 mm^2, respectively, while in TON-C, 83 + − 38/0.8 and 155 + −38/0.8 mm^2 of IgA- and IgG-bearing cells were detected, respectively. The IgA/IgG ratio was 0.71 + − 32 in TON-A, while 0.58 + − 38 in TON-C ($p < 0.05$). Therefore, TON-A exhibited a higher ratio of IgA- to IgG-bearing plasma cells than TON-C ($p < 0.01$).
